# *Kingella kingae* Osteoarticular Infections Approached through the Prism of the Pediatric Orthopedist

**DOI:** 10.3390/microorganisms10010025

**Published:** 2021-12-24

**Authors:** Giacomo DeMarco, Moez Chargui, Benoit Coulin, Benoit Borner, Christina Steiger, Romain Dayer, Dimitri Ceroni

**Affiliations:** Pediatric Orthopedics Unit, Pediatric Service of Surgery, University Hospitals of Geneva, CH-1211 Geneva, Switzerland; giacomo.demarco@hcuge.ch (G.D.); moez.chargui@hcuge.ch (M.C.); benoit.coulin@hcuge.ch (B.C.); benoit.borner@hcuge.ch (B.B.); christina.steiger@hcuge.ch (C.S.); romain.dayer@hcuge.ch (R.D.)

**Keywords:** *Kingella kingae*, osteoarticular infection, septic arthritis, acute hematogenous osteomyelitis, subacute osteomyelitis, surgical procedure, real time PCR, antibiotic treatment

## Abstract

Nowadays, *Kingella kingae* (*K. kingae*) is considered as the main bacterial cause of osteoarticular infections (OAI) in children aged less than 48 months. Next to classical acute hematogenous osteomyelitis and septic arthritis, invasive *K. kingae* infections can also give rise to atypical osteoarticular infections, such as cellulitis, pyomyositis, bursitis, or tendon sheath infections. Clinically, *K. kingae* OAI are usually characterized by a mild clinical presentation and by a modest biologic inflammatory response to infection. Most of the time, children with skeletal system infections due to *K. kingae* would not require invasive surgical procedures, except maybe for excluding pyogenic germs’ implication. In addition, *K. kingae*’s OAI respond well even to short antibiotics treatments, and, therefore, the management of these infections requires only short hospitalization, and most of the patients can then be treated safely as outpatients.

## 1. Introduction

Osteoarticular infections (OAI) represent serious affections, which may perturb subsequent bone development and may have severe consequences for articular function [1]. For pediatric orthopedists, implementing the proper etiology of OAI is crucial to confirm the diagnosis, to prescribe adjusted antibiotic therapy [2], and to improve the outcome [3,4,5]. The prognostic value also regards the epidemiological vigilance as identifying the causative pathogen has public health and infection control ramifications. An assortment of microorganisms has been identified, and, for many decades, *S. aureus* was considered the most prevailing pathogen of OAI in the pediatric population [6,7,8,9,10,11]. However, one realizes now that this superficial vision of the epidemiology of OAI in pediatric populations has overshadowed, for a long-time, the improvement in the knowledge concerning the cause and epidemiology, as well as developments in the diagnosis and therapy, of OAI. Better recognition of the microbiological causes of OAI has evolved dramatically in recent years. It is currently recognized that the clinical and biological aspects of OAI are closely related to the child’s age and to the incriminated pathogen. Any factors, such as the socioeconomic conditions, possible comorbidities, immune and vaccination status, changes in patterns of immunomodulating diseases, and the emergence of resistant bacteria, also need to be considered [12]. Thus, the pediatric orthopedist has seen the paradigm of the treatment of osteoarticular infections changing drastically during the last 10 years, and he, therefore, has had to adapt to a new protocol of care. This present review will, therefore, aim to establish the new concepts that revolve around *Kingella kingae* (*K. kingae*) infections and to define a specific treatment for this microorganism.

## 2. Historical Considerations

For many decades, the diagnosis of OAI in children was established through classical culture-based diagnostic methods, the pathogens searched for being bacteria, mycobacteria, fungi, and, more rarely, parasites. In this regard, it should be emphasized that negative cultures were frequently observed in pediatric OAI when using culture-based diagnostic tests, and this even occurred when proper samples were obtained. In fact, it is recognized that 21% to 70% of septic arthritis (SA) and 24% to 68% of acute hematogenous osteomyelitis (AHO) cases remained culture-negative despite meticulous attempts at identifying the causative pathogen [13,14,15,16]. In the situation of culture-negative OAI, the right therapeutic decision was more difficult to make and was usually based on empirical treatment without exact knowledge of the pathogen. This situation remained extremely frustrating for pediatric orthopedists who were forced and constrained to solely establish their treatment based on epidemiological studies of culture-positive infections. In addition, this situation seriously prejudiced studies interested in the bacterial etiology of OAI and in their prevalence. Nowadays, cultures still confer any advantages on non-culture-based direct detection methods since a pathogen detected by classical cultures is accessible for testing antimicrobial susceptibility. Thereby, any germ that is growing and is isolated from a culture gives a reliable result; the positive culture must be considered to have an undeniable specificity.

## 3. Improvement of the Microbiology Techniques

During the last three decades, the rapid development and the extended spread of new screening tests allow the better identification of bacteria and improve the recognition of infectious disease. Some tests explored pathogens’ antigens by immunochemical techniques, whereas other assays were developed to detect microbial nucleic-acid sequences. Since the early 2000s, nucleic acid amplification assays (NAAAs) gave bacteriologists the opportunity to detect infinitesimal quantities of bacterial DNA and provided clinicians with an effective and potent set of tools for detecting traces of bacteriological agents in clinical samples. During the last few years, the technology of NAAAs made effective progress, and continuing improvements were recorded both in terms of efficiency (development of primers amplifying species-specific targets by comparison with the universal primers) and specificity (considering the fact that the assays were less prone to contamination) [17]. It is now possible, by using NAAAs, to detect in clinical samples either nucleic acid sequences or specific antigens. Consecutively, NAAAs have become gradually indispensable due to the unavoidable restrictions of most of the culture-based diagnostic methods (e.g., time-consuming, technical complexity, bad sensibility with any fastidious organisms, and expense). Initially, these modern screening tests were intended to overcome the shortcomings of the classical culture methods for determining the etiology of infection. However, with the passage of time, NAAAs tended to replace completely classical culture methods and not only in selected classical culture methods. The crucial contribution of these diagnostic assays resulted in a drastic improvement in the competence of clinicians to reliably recognize infectious diseases. In this regard, the wide-scale use of NAAAs has remarkably improved the recognition of OAI, and thus changed both the incidence of pediatric OAI and their actual bacteriologic epidemiology [3,4,5]. Thus, the pediatric orthopedist can now know the germ responsible for most OAI that remained culture-negative before the advent of NAAAs.

More recently, the development and application of next generation sequencing (NGS) has constituted the starting point for the establishment of more efficient techniques in identifying microorganisms [17]. NGS is a high-throughput technique that makes possible the fast sequencing of the base pairs in entire genomes or in targeted regions of DNA or RNA samples. This technology offers ultra-high throughput, scalability, and speed; it has filled the gap of missing information beyond the capacity of traditional DNA sequencing technologies. NGS is a targeted sequencing technology that enables the rapid sequencing of every genome present in a clinical sample, obtaining millions of DNA strands, and thus reducing the requisite for the traditional cloning methods used in previous genome sequencing techniques. NGS has become indispensable in microbiology since it has replaced, with its genomic definition of pathogens, the conventional characterization of pathogens by staining properties, study of morphology, and metabolic criteria. The characterization of the pathogens’ genomes better defines what they are and may harbor crucial information about drug sensitivity; in addition, this can provide some insights regarding the relationship of different pathogens with others, which can be used to trace sources of infection outbreaks. Thus, the NGS method will arbitrarily amplify all the germs present in a clinical sample, allowing the detection of all potential pathogens.

However, NGS has the unfortunate defect of amplifying all nucleic acids, including, notably, host nucleic acids. Therefore, millions of reads will prove to be necessary in order to detect and identify only the pathogens of interest; this fact is still constituting the major hurdle that has slowed up the large-scale implementation of this technology in microbiological laboratories. In addition, deep sequencing requires the use of a larger number of reagents, and the evaluation of such extensive data imposes the development of an elaborate database. We can expect that NGS will move towards a more focused amplification of specific genomic regions of interest instead of massive simultaneous parallel sequencing as soon as the pathogens most frequently incriminated in OAI are recognized. Thus, this future more selective sequencing will end up achieving better specificity and improved sensibility and faster identification of suspected pathogens, thereby reducing the overall costs of the investigations [17].

## 4. New Bacteriological Reality in Osteoarticular Infections

From the 2000s, the number of cases of osteoarticular infections attributed to *K. kingae* has increased drastically. The dramatic increase in the detected cases must be credited to the widespread use of nucleic acid amplification assays. The use of NAAAs has provided irrefutable evidence that *K. kingae* has become the most common pathogen responsible for osteoarticular, infection in children aged less than 4 years [3,4,5,18]. This verdict has, above all, highlighted the necessity to use specific real-time quantitative polymerase chain reaction (qPCR) assays targeting *K. kingae* to detect, more efficiently, OAI caused by this pathogen [19]. Currently, *K. kingae* must be considered as the main germ of hematogenous infections of bones, joints, intervertebral discs, and tendon sheaths in children aged between 6 and 48 months. This gives a glimpse into the role of *K. kingae* in osteoarticular infection, which has been underestimated in the past [3,4,5]. The current evidence indicates that most OAI caused by *K. kingae* occur in children less than 4 years of age; this specific period of children’s life corresponds to the period of maximal oropharyngeal colonization by the germ. It is also recognized that intimate contact among infants and preschool-age children, as occurs in group childcare, represents the major risk factor for invasive infections due to *K. kingae* [20]. In addition, it is demonstrated that the maternal immunity conveyed to the fetus during pregnancy diminishes gradually during the above-mentioned period, above all between 6 and 24 months. Longitudinal investigations have proven that the average IgG levels against *K. kingae* are elevated at birth; then, they slowly decrease, reaching the lowest point at 6 to 7 months postnatally ^3^. Nevertheless, the IgG levels remain at low values until the age of 18 to 24 months, at which point a progressive increase in the serum levels of the immunoglobulin is noted [21,22]. Therefore, in the period between 6 and 48 months, a child is more prone to sustain invasive infections, such as OAI, due to *K. kingae*.

## 5. Prevalence of *Kingella kingae* in Pediatric Osteoarticular Infections

A few studies have reported on bacteriologically confirmed OAI in children; these reports may be easily interpretable, and most of them can be examined to summarize the frequency of *K. kingae* OAI among children of all ages, or when specifically considering children aged below 4 years. The main point that emerges from these studies is that around half of all OAI confirmed microbiologically in children of all ages are due to *K. kingae.* In fact, five studies have indicated that between 47.8% and 51% of microbiologically confirmed AOI are due to *K. kingae* whatever the age of the children [3,4,5,19,23]. Many studies, then, focused specifically on OAI in children aged between 6 and 48 months; these reports evidenced that, with the use of appropriate PCR assays, *K. kingae* was unquestionably the major bacterial cause of pediatric OAI in this specific age population. In fact, *K. kingae* is currently recognized to be the responsible pathogen for 52 to 93.8% of microbiologically confirmed OAI [3,4,5,19,23,24,25]. Thus, the time has come to stop believing that *S. aureus* remains the most common infective bacterial pathogen for OAI in all age groups since it has largely been replaced worldwide by *K. kingae*, especially in children less than 4 years of age [3,4,5,19]. However, it should be noted that *S aureus* remains the pathogen most frequently implicated in OAI in children over 4 years old and among adolescents.

## 6. Septic Arthritis Due to *Kingella kingae*

Septic arthritis is undeniably the most common form of osteoarticular disease of *K. kingae*. In fact, some reports demonstrated that septic arthritis represents between 53 and 82.8% of all OAI due to this pathogen [26,27,28]. *K. kingae* septic arthritis usually involves large weight-bearing articulations, such as the hip, knee, ankle, shoulder, or elbow [20,25,27,28,29,30,31,32]. In terms of frequency, the infection tends to involve the lower extremity the most, and the knee is the joint that is the most frequently incriminated. However, atypical joints, such as the sternoclavicular, acromioclavicular, tarsal, or metacarpophalangeal/metatarsophalangeal joints, are overrepresented in *K. kingae* arthritis compared with septic arthritis caused by other pathogens [20,25,27,28,29,30,31,32]. The clinical and biological aspects of septic arthritis can, unfortunately, be truncated when *K. kingae* is responsible for infection [33]. The synovial fluid examination in children with culture-proven arthritis due to *K. kingae* demonstrated low leukocyte counts in a substantial number of cases. On that point, the recognized cut-off value of 50,000 WBC/mL in synovial fluid aspirates, used as a diagnostic factor defining bacterial arthritis, may erroneously exclude the diagnosis of *K. kingae* arthritis and should, therefore, be used with great caution [33]. In fact, a few studies demonstrated that the WBC count of the synovial fluid showed less than 50,000 WBC/mL in a quarter of the cases, and that and the examination of the Gram-stain is, most of the time, negative [21,22,34]. Thus, the afebrile presentation, the mild clinical symptoms, and the absence or the little disturbance of acute phase reactants in *K. kingae* arthritis do not meet the diagnostic criteria of a septic joint [35]. Even worse, when using the application of Kocher’s predictive algorithm [36], it seems that three-quarters of children with culture-proven *K. kingae* septic arthritis of the hip would have been considered to have transient synovitis [35]. The clinical experience has taught us that this algorithm should not be used for children less than 4 years old. Here again, the pediatric orthopedist must be extremely vigilant in the face of clinical situations not very suggestive of arthritis.

Finally, the clinical course of septic arthritis due to *K. kingae* is very different from those of arthritis caused by pyogenic microorganisms, which stimulate a huge influx of neutrophils to the site. In septic arthritis due to pyogenic pathogens, the clinical course is characterized by a potent activation of the immune response, in association with high levels of cytokines and reactive oxygen species, and an increase in the release of host matrix metalloproteinases and other collagen-degrading enzymes, which, in conjunction with bacterial toxins, lead to joint cartilage destruction [37,38,39]. In addition, the antigen-induced inflammatory response may persist and continue to damage the joint architecture even after the infection has been cleared [38,39]. Fortunately, the clinical experience acquired during the management of OAI with *K. kingae* seems to demonstrate that arthritis due to this particular pathogen does not follow this pattern of cartilage destruction. Therefore, many cases of *K. kingae*’s septic arthritis may even be treated by a simple syringe injection–aspiration procedure, and thus, especially when the leukocyte count appears low in the synovial fluid examination, the attitude will not be defensible in pyogenic septic arthritis.

## 7. *K. kingae* Osteomyelitis

The anatomical sites affected by *K. kingae* osteomyelitis include long bones, such as the femur, tibia, humerus, radius, and ulna [3,4,5,19,21,22]. Nevertheless, any bone rarely infected by other pathogens, such as the sternum, the clavicle, the talus, or the calcaneum, may also be affected by *K. kingae* osteomyelitis [3,4,5,19,30]. *K. kingae* osteomyelitis represents between 15 to 31% of all OAI due to this pathogen, and more than a quarter of such osteomyelitis is concomitant with septic arthritis [3,5].

It is also interesting to highlight that epiphysis or apophysis, which are almost never affected by other organisms, may be frequently involved in *K. kingae* osteomyelitis [40,41]. Some reports have suggested that patients with isolated osteomyelitis had a more prolonged duration of symptoms before admission and presented with a lower body temperature compared with children with septic arthritis alone [30]. Without an appropriate treatment, acute osteomyelitis can sneakily evolve into subacute osteomyelitis. Subacute osteomyelitis is an atypical osteomyelitis that is most likely attributed to an atypical host–pathogen relationship that may be explained by any combination of increased host resistance, decreased virulence of the causative pathogen, and/or prior antibiotic exposure [42,43,44,45,46]. In most cases, culture-based methods fail to identify the causative organism, especially when fine-needle aspiration is performed. Surgical drainage may yield positive cultures in 40% to 75% of patients [41]. Subacute osteomyelitis can be divided into two main clinical forms according to the children’s age and to its bacteriological etiology [41]. The first form, called the infantile form, affects children aged between 6 months and 4 years. Approximately 90% of all PSAHO affect patients in this age group, with *K. kingae* as the main observed microorganism [41]. In these young children, the clinical course is most likely attributable to the natural low virulence of *K. kingae*. Many children in this age group are usually recognized late as having an osteoarticular infection, and the accurate diagnosis is generally delayed after a bony lytic lesion has occurred [40,41]. The second form, entitled the juvenile form, affects children older than 4 years and *S. aureus* appears as the major responsible pathogen. Subacute osteomyelitis due to *K. kingae* follows a benign course, and the recommended treatment for sub-acute osteomyelitis, when radiographic investigations demonstrate lucent lesions or nidus, is curettage, biopsy, and culture, followed by antibiotics [40,41,47]. However, a few authors have suggested that antibiotics alone may be adequate and that surgery should be reserved only for “aggressive lesions”, as well as those that do not respond to antibiotics [40,41,47]. However, it is generally agreed that treatment should not be initiated until proper drainage and bacteriological samples have been obtained [40,41,47].

## 8. Other Atypical Osteoarticular Infections Caused by *K. kingae*

Excepting arthritis and osteomyelitis, spondylodiscitis is probably the main frequent form of invasive *K. kingae* infection in children less than 4 years old. Childhood spondylodiscitis is a term frequently used to describe a continuum of spinal infections, from discitis to spondylodiscitis as well as vertebral osteomyelitis with occasional associated soft tissue abscesses. Three main clinical forms of spondylodiscitis have been described in the pediatric population according to the age of the patient [48,49,50]. The neonate form affects infants under 6 months and is the most serious manifestation of the disease, often presenting with *Staphylococcus aureus* septicemia and multiple infectious foci. The infantile form affects children from 6 months (end of maternally derived immunity) to 48 months of age, and this age group represents 80% of childhood spondylodiscitis. In this group, some studies have suggested that *K. kingae* could be the most frequent microorganism responsible for the spinal infection [48,49,50]. Finally, in the third form, affecting children older than 4 years, patients are more prone to be febrile and ill-appearing and to sustain vertebral osteomyelitis due to *S. aureus.* This triphasic age distribution is currently widely used among pediatric orthopedists since it explains both the different microbiologic epidemiology and the different clinical forms among the age groups.

Invasive *K. kingae* infections can also give rise to atypical osteoarticular infections, such as cellulitis, pyomyositis, bursitis, and tendon sheath infections [31,51,52]. Thus, we suggest using and incorporating MRI into modern diagnostic algorithms for OAI to better identify unusual locations of OAI.

## 9. Clinical and Biological Aspects of OAI Due to *Kingella kingae*

The presentation of *K. kingae* OAI is often characterized by a mild clinical presentation and by a moderate biologic inflammatory response to infection, with the consequence that these children present few, if any, criteria evocative of OAI. In fact, most of the children with OAI due to *K. kingae* appear in excellent general condition; they usually present with symptomatology suggestive of muskulosketelal disease with either a member saving, a limp, or a refusal to bear weight. Signs on physical examination will revolve around localized pain, limited articular range of motion, or non-traumatic effusion. However, it may be very difficult to determine, in some cases, even where the epicenter of pain is.

In addition, only 10 to 33% of children with OAI caused by *K. kingae* have a body temperature ≥38 °C at admission [3,18,19,21,22,24]. Recent reports about large series demonstrated that fever was absent in between 71 and 74% of children with proven OAI due to *K. kingae* [3,53]. Most of the patients have normal or near normal white blood cell counts and C-reactive protein levels. In the largest series about OAI due to *K kingae*, a blood WBC count <15,000 cells/mm^3^ was found in 51% of patients in whom the data were available [21]. In a recent Swiss study about 151 patients with OAI due to *K. kingae*, the mean blood WBC count was 11.9 × 10^9^/L, and the absolute value was considered as elevated in only 11.2% of the cases [3]. In the same study, the CRP level was elevated (>10 mg/L) in 61% of the patients, but the median CRP value of all the cases was only 15.5 mg/L [3]. A few studies suggested that the erythrocyte sedimentation rate and platelet counts seem to be the most sensitive markers of inflammation when a *K. kingae* OAI is present [3,4,5,19].

Different models to allow the differentiation of *K. kingae* OAI from those due to typical pathogens, in children aged less than 4 years of age, have been described. The first model was built around the following four parameters: temperature at admission <38 °C; C-reactive protein <55 mg/L; white blood cell count <14,000 leukocytes/mm^3^; and band shift <150 forms/mm^3^ [54]. In another study, the same authors tried to define a model for accurately distinguishing between MSSA and *K. kingae.* The model in question to predict *K. kingae* OAI included the following cut-offs for each parameter: age <43 months, temperature at admission <37.9 °C, CRP <32.5 mg/L, and platelet count >361,500/mm^3^ (Coulin & Ceroni, to be published).

## 10. Radiologic Investigations for *K. kingae* OAI

The pediatric orthopedists have now learned that osteomyelitis due to *K. kingae* could have a very particular radiological presentation because of the low virulence of this pathogen. It is currently recognized that osteomyelitis caused by *K. kingae* may have a more prolonged duration of symptoms before admission and present with a lower body temperature compared with children with osteomyelitis due to pyogenic pathogens. Thus, osteomyelitis due to *K. kingae* can easily be unrecognized or even trivialized at first and sneakily evolve into subacute osteomyelitis. Radiologically, this subacute osteomyelitis may appear in the form of lytic lesions. It is also interesting to highlight that the epiphysis or apophysis, which are almost never invaded by other organisms, may be commonly involved in *K. kingae* osteomyelitis [40,41]. In this regard, it seems obvious for pediatric orthopedists that any lytic lesions occurring in a child under 4 years old will be very suggestive of *K. kingae* subacute osteomyelitis [3,4,5,19].

On the other hand, it is widely accepted that early MRI may be helpful in the investigation and management of OAI. It is reliable for assessing the viability and blood flow to the infected bone, for identifying bone and soft tissue abscesses needing surgical drainage, for detecting joint effusion or distention of the capsule, and for directing biopsies. MRI is also useful in the diagnosis of arthritis, and it is able to discriminate between different types of arthritis [55]. Clinical experience has taught pediatric orthopedists that MRI is even more important in the investigation of *K. kingae* infections. In fact, OAI caused by *K. kingae* may be quite heterogeneous and can take very different forms, such as isolated arthritis, osteomyelitis, concomitant arthritis and osteomyelitis, muscle abscess, or tendon sheath infection. Even if OAI due to *K. kingae* appear clinically less severe, using and incorporating MRI into modern diagnostic algorithms for these OAI should be considered to better identify the atypical locations of these particular and atypical infections. Interestingly, it has been demonstrated that MRI has a high accuracy in discriminating OAI due to *K. kingae* from those caused by gram positive cocci [56]. Mild soft tissue reaction, absence or mild bone reaction, and epiphyseal cartilage involvement are findings suggesting *K. kingae* rather than gram positive cocci. Since MRI is frequently performed before bacteriological results become available, image-based diagnosis may be a useful complementary tool in differentiating OAI caused by *K. kingae* from OAI caused by more aggressive organisms.

## 11. Therapeutics Strategies for the Management of OAI Due to *K. kingae*

Several controversies concerning the treatment of OAI are still contributing to the debate, and this debate seems even more relevant when osteoarticular infections due to *K. kingae* must be treated. In fact, there is, nowadays, no consensus about the treatment of OAI considering which infections may be treated medically and which will need a surgical approach.

The clinical presentation and outcome may be vastly different when considering the microbiological causes of OAI, and, thus, the required treatment can be diametrically opposed from one case to another. For example, OAI due to *S. aureus* present a more severe course of disease, a slower clinical response, and a potentially worse outcome, requiring, most of the time, invasive diagnostic and therapeutic procedures and rapid antibiotic treatment [54,57], whereas many OAI due to *K. kingae* could be theorically treated only with antibiotherapy [3].

Thus, the main question to be answered, especially when treating a child with an OAI due to *K. kingae*, is “does the child need surgery?” [3,4,5]. Most of the time, there are three basic reasons for proposing a surgical intervention in many bone and joint infections: i.e., microbiologic diagnosis, infectious source control, and preservation of maximal function [3,4,5]. Starting with microbiologic diagnosis, identification of the pathogen responsible for infection would be extremely helpful for tailoring definitive antibiotic treatment regarding the choice, the route, and the duration for antibiotic therapy.

Theoretically, children with skeletal system infections due to *K. kingae* would not require invasive surgical procedures, except maybe for excluding pyogenic germs’ implication. On that point, a diagnostic procedure has been developed to avoid unnecessary surgical procedures with a bacteriological diagnostic aim. It has been suggested that the performance of a sensitive *K. kingae*-specific NAAA on an oropharyngeal specimen may provide strong evidence that this microorganism is responsible for OAI [58]. However, it should be kept in mind that a positive test is not an irrefutable proof of the etiology of the disease since around 10% of young children carry the organism. Contrariwise, a negative PCR result rules out *K. kingae* as the causative pathogen of OAI [58].

## 12. Antibiotic Treatment for OAI Due to *K. kingae*

Most of the patients with *K. kingae*’s OAI respond promptly to conservative treatment with appropriate antibiotics to such an extent that the antibiotic treatment could be reasonably shortened. In many cases, any patients with *K. kingae*’s OAI may follow an abortive course, even when no antimicrobials therapy is administered. Nowadays, there is still a lack of controlled studies that permit the formulation of evidence-based recommendations on the most efficient antibiotic, on the necessity or not to investigate production of beta-lactamase, and on the optimal length of therapy for *K. kingae* OAI [32]. Drug therapy for osteoarticular infections usually consists of the intravenous administration of oxacillin/nafcillin or a second- or third-generation cephalosporin while pending culture results [18,19,59]. In areas in which community associated methicillin-resistant *Staphylococcus aureus* is prevalent and when the clinical presentation is acute, a combination of a b-lactam antibiotic and vancomycin may be suggested [60]. The clinical response is then used to guide switching to oral antibiotics (usually within less than 3 days) The current trend is to shorten as much as possible the length of antibiotic treatment since it is noted that *K. kingae*’s OAI respond well even to short antibiotics treatments. Antibiotic treatment generally varies from 2 to 3 weeks for *K. kingae* arthritis, from 3 to 6 weeks for osteomyelitis, and from 3 to 12 weeks for spondylodiscitis [30]. However, the current trend is to shorten as much as possible the length of antibiotic treatment since it is noted that *K. kingae’s* OAI respond well even to short antibiotics treatments. In fact, many authors consider that OAI due to *K. kingae*, characterized by a subacute course and low bacterial concentrations, could be treated solely by shorter oral antibiotic therapy [3,4,5].

## 13. Antibiotic Susceptibility of *K. kingae*

During the last few years, the susceptibility of *K. kingae* to antibiotics that are generally given to children with suspected or confirmed invasive infection has been greatly studied, and this microorganism’s characteristics are currently better recognized. *K. kingae* is considered to be extremely susceptible to ampicillin, penicillin, second- and third-generation cephalosporins, cotrimoxazole, ciprofloxacin, macrolides, tetracycline, and chloramphenicol [30,61]. The organism exhibits reduced susceptibility to oxacillin and clindamycin [62,63] and appears totally resistant to vancomycin and trimetoprim [62,63,64,65,66,67]. Some reports have described occasional in vitro resistance to cotrimoxazole, erythromycin, and ciprofloxacin [62,63,64,65,66,67]. Yagupsky et al. have demonstrated that 38.5% of the isolates from their healthy respiratory carriers and patients with invasive infection enclosed *K. kingae* strains that were resistant to clindamycin [62,63]. More alarming is the discovery of strains producing b-lactamase recovered from an HIV-positive patient in the USA [68] and in three isolates from children in Iceland [69]. The culture of *K. kingae* remains suboptimal if not, frankly, ineffective, even when the samples from infected joint or bone are inoculated directly into blood culture vials.

Because of the large-scale use of nucleic acid amplification assays, the susceptibility of the organism to antimicrobial drugs administered to children with invasive infection due to *K. kingae* is being less frequently or not at all investigated. Therefore, clinicians will not have any information about the antibiotic susceptibility of the organism in many clinical situations, and there is the potential risk to treat patients affected with an antimicrobial-resistant organism infection ineffectively. On this subject, a few studies demonstrated that the strain of *K. kingae* isolated in the throat was responsible for OAI; this observation highlighted the possibility of isolating with significant effectiveness the *K. kingae* strain responsible for OAI using throat swabs [58,70]. Thus, there is a real interest to perform throat swabs both to isolate the bacteria and study its antimicrobial sensitivity profile.

## 14. Functional Prognosis of OAI Due to *K. kingae*

OAI due to *K. kingae* have a good character: they are considered as benign with a mild-to-moderate clinical presentation, they have a favorable prognosis after adequate antibiotic treatment, and they seldom lead to long-term sequelae [18,21,22,32,57]. There is a world of difference between infections due to pyogenic germs and those caused by *K. kingae*. On that point, the clinical course is usually better for children with OAI caused by *K. kingae*, as evidenced by shorter hospitalization and fewer adverse events [54,57]. This can be explained both by the low virulence of *K. kingae* [22] and by its high susceptibility to β-lactam antibiotics [22,30,32].

However, the initial benign clinical presentation of *K. kingae* may result in delayed diagnosis [30,32,54] and may thus lead to subsequent severe infectious bone lesions [71]. In fact, the mildness of the clinical signs during *K. kingae* osteomyelitis may lead to a diagnostic delay with a more destructive nature of the lesions, presence of intraosseous abscesses, and potential damage to the growth cartilage. Such subacute osteomyelitis is rare and often misdiagnosed because of insidious symptoms [43,44,45,72]. Fortunately, such complicated cases are rare (less than 5% of *K. kingae* osteoarticular infections), and the functional results are quite satisfactory even in the case of physeal damage [71]. Even if the emergency character is not the same as for pyogenic infections, care must be taken, and antibiotic treatment should be introduced as soon as the diagnosis of OAI due to *K. kingae* is suspected.

## 15. Conclusions

Advances in molecular sequencing technologies have revealed new perspectives in the field of OAI diagnoses. *K. kingae* is currently considered as the major bacterial cause of OAI in children younger than 48 months, and this is the case in most countries worldwide. The presentation of *K. kingae* OAI is often characterized by a mild clinical presentation and a moderate biologic inflammatory response to infection, with the consequence that these children present few, if any, criteria evocative of OAI. *K. kingae*’s OAI entail different degrees of urgency for therapeutic management and for the need of a surgical procedure than those due to pyogenic pathogens. Theoretically, children with skeletal system infections due to *K. kingae* would not require invasive surgical procedures, except maybe for excluding pyogenic germs’ implication. Thus, these elements have significantly modified the paradigm of the management of OAI caused by *K. kingae*. The current trend is to shorten, as much as possible, the length of antibiotic treatment since it is noted that *K. kingae*’s OAI respond well even to short antibiotics treatments. Thus, the initial management of these infections requires only short hospitalization, and most of the patients can then be treated safely as outpatients. Finally, OAI due to *K. kingae* have a favorable prognosis after adequate antibiotic treatment, and they seldom lead to long-term sequelae.
